# Stavudine Toxicity in Adult Longer-Term ART Patients in Blantyre, Malawi

**DOI:** 10.1371/journal.pone.0042029

**Published:** 2012-07-26

**Authors:** Joep J. van Oosterhout, Jane Mallewa, Symon Kaunda, Newton Chagoma, Yassin Njalale, Elizabeth Kampira, Mavuto Mukaka, Robert S. Heyderman

**Affiliations:** 1 Malawi-Liverpool Wellcome Trust Clinical Research Programme, University of Malawi College of Medicine, Blantyre, Malawi; 2 Department of Medicine, University of Malawi College of Medicine, Blantyre, Malawi; 3 Department of Pathology, University of Malawi College of Medicine, Blantyre, Malawi; 4 Division of Human Genetics, Faculty of Health Sciences University of Cape Town, Cape Town, South Africa; University of New South Wales, Australia

## Abstract

**Background:**

Stavudine is an effective and inexpensive antiretroviral drug, but no longer recommended by WHO for first-line antiretroviral regimens in resource-limited settings due to toxicity concerns. Because of the high cost of alternative drugs, it has not been feasible to replace stavudine in most adults in the Malawi ART programme. We aimed to provide policy makers with a detailed picture of stavudine toxicities in Malawians on longer-term ART, in order to facilitate prioritization of stavudine replacement among other measures to improve the quality of ART programmes.

**Methods:**

Prospective cohort of Malawian adults who had just completed one year of stavudine containing ART in an urban clinic, studying peripheral neuropathy, lipodystrophy, diabetes mellitus, high lactate syndromes, pancreatitis and dyslipidemia during 12 months follow up. Stavudine dosage was 30 mg irrespective of weight. Cox regression was used to determine associations with incident toxicities.

**Results:**

253 patients were enrolled, median age 36 years, 62.5% females. Prevalence rates (95%-confidence interval) of toxicities after one year on stavudine were: peripheral neuropathy 21.3% (16.5–26.9), lipodystrophy 14.7% (2.4–8.1), high lactate syndromes 0.0% (0–1.4), diabetes mellitus 0.8% (0–2.8), pancreatitis 0.0% (0–1.5). Incidence rates per 100 person-years (95%-confidence interval) during the second year on stavudine were: peripheral neuropathy 19.8 (14.3–26.6), lipodystrophy 11.4 (7.5–16.3), high lactate syndromes 2.1 (0.7–4.9), diabetes mellitus 0.4 (0.0–1.4), pancreatitis 0.0 (0.0–0.2). Prevalence of hypercholesterolemia and hypertriglyceridemia increased from 12.1% to 21.1% and from 29.5% to 37.6% respectively between 12 and 24 months. 5.5% stopped stavudine, 1.3% died and 4.0% defaulted during follow up. Higher age was an independent risk factor for incident peripheral neuropathy and lipodystrophy.

**Conclusion:**

Stavudine associated toxicities continued to accumulate during the second year of ART, especially peripheral neuropathy and lipodystrophy and more so at increasing age. Our findings support investments for replacing stavudine in first-line regimens in sub-Saharan Africa.

## Introduction

In 2004 Malawi successfully embarked on an antiretroviral therapy (ART) scale-up programme with a public health approach. Important characteristics of this programme are free treatment, standardized drug regimens, simplified and mainly clinical monitoring and promotion of decentralized clinical care [1], [2]. At the end of 2011 over 250,000 patients were receiving ART. Although a new first-line regimen without stavudine was introduced in 2011for certain priority groups, around 90% of patients still use the standard generic combination of stavudine, lamivudine and nevirapine [3].

Stavudine is inexpensive, potent, has a relatively high barrier to resistance and is available in a generic combination tablet. Experience in several African countries has shown that during the first stages of treatment stavudine can be used without laboratory monitoring for side effects. However, stavudine is no longer recommended in first-line ART regimens in affluent countries due to its unfavorable toxicity profile. Several studies have provided insight into the occurrence of stavudine related side effects in patients from sub-Saharan Africa. Peripheral neuropathy is one of the most common toxicities with prevalence rates that varied from 20 to 34% after 1 to 3 years in Uganda, South Africa and Kenya [4], [5]. Two studies showed a high prevalence of lipodystrophy in adult Rwandans on stavudine [6], [7]. High lactate syndromes are relatively rare, but appear to occur more frequently than in affluent countries and lactic acidosis carries a very high mortality [8]. Pancreatitis is another serious side effect associated with stavudine, which appeared to be uncommon in Ugandans [4], although cases may have been missed due to the retrospective design, the non-specific presentation and lack of diagnostic tests. Dyslipidemia, glucose intolerance and diabetes mellitus are common in western patients on ART, probably due to HIV infection, antiretroviral drugs as well as traditional risk factors. Because monitoring of lipid and glucose levels during ART is rarely feasible in sub-Saharan Africa, data from the region are sparse.

WHO has recently recommended that stavudine should be replaced in standard first-line ART regimens in resource limited settings, preferably with tenofovir [9]. In Malawi, this is being implemented for children, pregnant and breast feeding women and patients receiving TB treatment but replacing stavudine for all adult patients is currently unaffordable. Indeed, due to the large number of patients involved, the budgetary demands of introducing more expensive alternatives to stavudine are considerable [10] and may compete with other priorities to improve ART programmes such as extending ART eligibility and viral load monitoring for ART failure.

To assist policy makers in their decisions about the replacement of stavudine in the first-line regimen we have prospectively monitored the full burden of stavudine toxicities in a cohort of patients who had just completed one year of treatment in Blantyre, Malawi. We have also identified the risk factors for these stavudine toxicities.

## Methods

### Study site and subjects

The study took place at the ART clinic of Queen Elizabeth Central Hospital, in Blantyre, the commercial capital of the Southern region of Malawi. The clinic was established in 2000, started providing ART free of charge in 2004, and currently cares for around 9,000 patients on ART. We enrolled consecutive patients aged 15 years and above, living in the hospital catchment area, who had started ART at the clinic and had just completed one year of stavudine containing treatment. They were seen for routine ART care and study procedures at baseline (i.e. 12 months after ART initiation) and 3, 6, 9 and 12 months later. Patients were encouraged to visit the clinic in case of any intercurrent illness. We did not trace participants actively in the community if they did not attend for follow up visits.

### Data collection and diagnostic procedures

At enrolment demographic information and details of the ART history were recorded. A standard proforma for symptoms and physical examination was used. We diagnosed peripheral neuropathy on the basis of characteristic symptoms that had started after the initiation of ART. Due to lack of sophisticated imaging techniques to diagnose lipodystrophy, we used the Lipodystrophy Case Definition Study-based Questionnaire [11] at baseline and after six and 12 months. This instrument has been validated in African settings [7] and is a combination of patient self-assessment and inspection by the clinician of seven body areas (face, neck, chest, abdomen, arms, legs and buttocks). Body shape changes were scored as minimal, moderate or much. Fat loss and gain were distinguished. To diagnose pancreatitis, we investigated all new episodes of abdominal pain with an amylase serum level and abdominal ultrasonography. Lactate was measured at each visit with the hand-held Lactate Pro® (Arkray Europe B.V., Amstelveen, the Netherlands) at the point-of-care. Patients were rested and were checked for dehydration before sampling. Strongly elevated values were confirmed with a repeat test plus determination of an anion gap and venous CO2 level. We diagnosed the following high lactate syndromes: severe hyperlactatemia if lactate was repeatedly >5.0 mmol/L (reference range <2.2 mmol/L) without symptoms; symptomatic hyperlactatemia if lactate was repeatedly >5.0 mmol/L and symptoms associated with lactic acidosis were present and lactic acidosis if there were also manifestations of metabolic acidosis (acidotic breathing and/or reduced venous CO_2_ and an increased anion gap). A diagnosis of diabetes mellitus was based on WHO criteria [12]: if a random or fasting blood glucose was repeatedly higher than 200 mg/dL or 126 mg/dL respectively. A serum lipid spectrum was determined at enrolment and after 12 months (non-fasting) with the Beckman Coulter CX5 Pro (Beckman Coulter, Halfway House, RSA). Study participants experiencing peripheral neuropathy, lipodystrophy, a high lactate syndrome, pancreatitis and/or diabetes were labeled as “any stavudine toxicity”.

To obtain an overview of stavudine toxicities in the first year on ART, we retrospectively studied patient records of all adults who started ART at Queen Elizabeth Central Hospital during the same episode as the study participants but were not enrolled into the cohort. We collected patient characteristics at the start of ART, standardized ART outcomes and information about toxicity events leading to replacement of stavudine during the first year on ART.

### Statistics

Data were double entered into a secure Microsoft Access SQL database. For statistical analyses Stata for windows software (version SE/11; 4905; Stata corp; College Station, Texas 77845 USA) was used. Patient characteristics were compared with Chi-squared and t-tests. The prevalence rates at enrolment and incidence rates during follow-up, of high lactate syndromes, lipodystrophy, peripheral neuropathy, pancreatitis, dyslipidemia and diabetes mellitus were expressed with 95% confidence intervals. The probability of experiencing each of the toxicities at specific follow-up time points were estimated using Kaplan-Meier curves; between group comparisons were performed using log rank tests. Risk factors for incident toxicities were assessed using Cox proportional hazards regression models which included age, sex, body mass index (BMI), WHO stages, previous diagnosis of tuberculosis, estimated creatinine clearance, smoking and alcohol use. Diagnostic checks to assess the appropriateness of the proportional hazard assumptions were done using Schoenfeld residuals. A paired t-test was used to assess change in serum lipids levels between month12 and month 24 on ART. All tests were 2-sided and were tested at 5% level of significance.

### Ethical considerations

The study was approved by the College of Medicine Research and Ethics Committee (study number P.01/09/719) and written informed consent was obtained from all patients.

## Results

### Enrolment process

**Figure 1:**
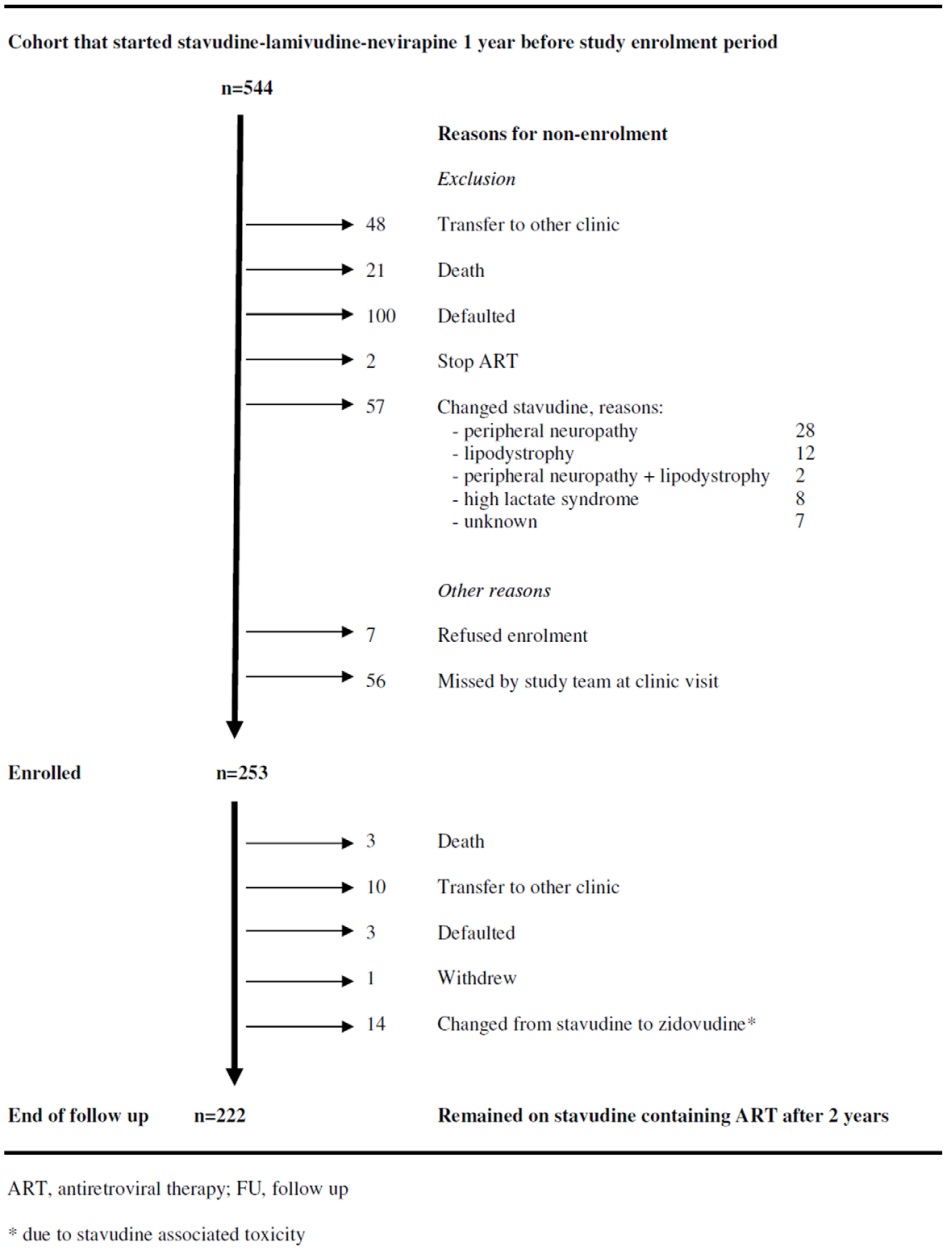
Study diagram.

From May through September 2009, we consecutively enrolled 253 patients who had just completed one year of ART into the prospective cohort study. These study participants originated from 544 adults who had started the standard first-line ART regimen (stavudine-lamivudine-nevirapine) at QECH from May through September 2008. The mortality, default and transfer-out rates for patients in the first year of routine ART care were 3.9%, 18.4% and 8.8% respectively. *Figure 1* shows attrition during the first year on ART and other reasons for not enrolling patients. Out of the 291 patients who were not enrolled in our prospective cohort, adequate information was available in the routine system of 190. When we compared these 190 patients to the study participants (n = 253), we found that mean age (36.3 vs. 36.6 years; p = 0.72), prevalence of female gender (58.5 vs. 62.5%; p = 0.38) and median CD4 count at ART initiation (141.0 vs. 141.0 cells/micro-liter; p = 0.69) were similar. However, study participants were less frequently in WHO stage 3 or 4 (41.9 vs. 60.3%; p<0.001).

### Patient characteristics

Patient characteristics in *table 1* show that a considerable percentage of study participants had had progressive clinical disease and/or advanced immune suppression when they started ART. The mean age of females was lower, fewer females had received tuberculosis treatment, fewer reported current alcohol consumption and they had a higher estimated creatinine clearance compared to males.

**Table 1:** Patient characteristics.

Characteristic	Females	Males	Total	P-value
**N**	158 (62.5)	95 (37.5)	253	
**Median age (IQR), years**	33 (29–40)	38 (34–46)	36 (31–43)	0.001
**Median BMI (IQR), kg/m^2^**	23.5 (20.9–26.1)	22.4 (21.2–24.6)	23.1 (21.2–25.5)	0.076
**BMI categories**				
underweight (<18.5 kg/m^2^)	7 (4.4)	3 (3.2)	10 (4.0)	0.073
normal (18,5–25 kg/m^2^)	98 (62.0)	72 (75.8)	170 (67.0)	
overweight (>25 kg/m^2^)	43 (27.0)	19 (20.0)	62 (25.0)	
missing	10 (6.0)	1 (1.0)	11 (4.0)	
**Median CD4 at start ART (cells/µL)**	155	114	141	0.28
0–199	101 (67.8)	65 (74.7)	166 (65.6)	0.524
200–349	43 (28.9)	20 (23.0)	63 (24.9)	
≥350	5 (3.4)	2 (2.3)	7 (2.8)	
missing	9 (5.7)	8 (8.4)	17 (6.7)	
**WHO stage at start ART**				
I	39 (24.7)	18 (18.9)	57 (22.5)	0.119
II	61 (38.6)	29 (30.5)	90 (35.6)	
III	42 (26.6)	39 (41.1)	81 (32.0)	
IV	16 (10.1)	9 (9.5)	25 (10.0)	
**Current smoker**	0	0	0	
**Current alcohol user**	1 (0.6)	6 (6.3)	7 (2.8)	0.008
**Previous TB treatment**	33 (20.9)	33 (34.7)	66 (26.1)	0.015
**Previous KS diagnosis**	3 (1.8)	3 (1.8)	6 (2.3)	0.524
**Median eCC (IQR), ml/min**	90.4 (74.5–104.7)	71.9 (63.5–82.6)	82.1 (70.1–98.7)	<0.001
**eCC categories**				
≥90	81 (52.3)	39 (41.1)	120 (49.9)	0.112
89–60	63 (39.9)	50 (52.6)	113 (44.7)	
59–30	8 (5.2)	2 (2.1)	10 (3.9)	
<30	4 (2.5)	4 (4.2)	8 (3.1)	
missing	4 (2.5)	4 (4.2)	4 (4.2)	
**On cotrimoxazole prophylaxis**	139 (88.1)	85 (89.5)	224 (88.5)	0.288
**ART regimen**				
Stavudine/lamivudine/nevirapine	145 (91.8)	89 (93.7)	234 (92.5)	0.576
Stavudine/lamivudine/other	13 (8.2)	6 (6.3)	19 (7.5)	

All characteristics are expressed as n (%) and are measured at enrolment, unless otherwise indicated.

All characteristics are at enrolment into the study (after one year on ART), except WHO stage and CD4 count as indicated.

IQR, inter-quartile range; BMI, body mass index; ART, antiretroviral therapy; WHO, world health organization; TB, tuberculosis; KS, Kaposi's sarcoma; eCC, estimated creatinine clearance (Cockroft-Gault method).

### Standard ART outcomes

During their second year on ART, three patients died (1.3%; 95%-CI 0.2–30.0), ten defaulted (4.0%; 95%-CI 2.0–6.6), three transferred to another clinic (1.3%; 95%-CI 0.2–30.0), one withdrew from the study and none stopped ART during follow up (*figure 1*). No patient started a second-line regimen due to ART failure.

### Stavudine related toxicities

**Figure 2:**
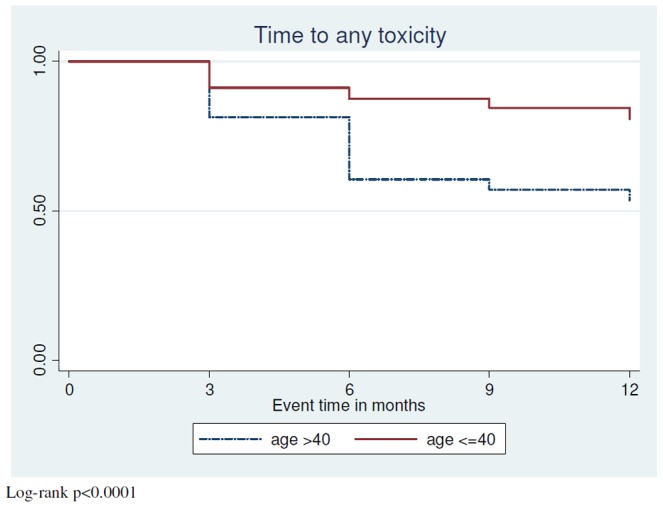
Time to first stavudine toxicity (peripheral neuropathy, lipodystrophy, high lactate syndrome, pancreatitis or diabetes) in patients starting the second year of stavudine containing ART without any of these toxicities.

At enrolment, i.e. after one year on stavudine containing ART, side effects were common, in particular peripheral neuropathy and lipodystrophy. Incidence rates of peripheral neuropathy and lipodystrophy in the second year on ART were high, but there were few new high lactate syndromes and diabetes diagnoses and no case of pancreatitis (*table 2*). Time to first stavudine toxicity in patients free from side effects at enrolment is indicated in *figure 2*, showing that the time to first toxicity was significantly shorter in those aged younger than 40 years (log-rank p<0.0001). There was good agreement between patients and investigators in the assessment of the presence of lipodystrophy; discrepancies occurred only in 16 out of 732 assessments (2.2%). Where discrepancies occurred, six times patients noted changes that were not observed by investigators and ten times vice versa. Of the patients who had lipodystrophy, 27.3% had lipo-hypertrophy, 39.4% had lipo-atrophy and 33.3% had both. The self-reported severity of lipodystrophy was as follows: 10.3% severe, 37.9% moderate and 51.7% mild, and 96.4% said it had no impact on adherence.

**Table 2:** Prevalence rates at enrolment and incidence rates during 12 months of follow up of stavudine associated toxicities in 253 Malawian adults.

Toxicity	Prevalence Rate*		Incidence Rate	
	PR (%)	95%-CI	IR (/100 py)	95%-CI
**Peripheral neuropathy**	21.3	16.5–26.9	19.8	14.3–26.6
**Lipodystrophy**	14.7	2.4–8.1	11.4	7.5–16.3
**Pancreatitis**	0	0–1.5	0	0.0–0.2
**Diabetes mellitus**	0.8	0–2.8	0.4	0.0–1.4
**High lactate syndrome** †	0	0–1.4	2.1	0.7–4.9
**Increased TC (>200 mg/dL)**	11.9	8.1–16.4	9.9	7.9–13.3
**Increased TG (>110 mg/dL)**	5.5	3.1–9.1	13.9	9.2–16.7
**Any toxicity** ¶	22.9	17.8–28.6	26.7	20.1–34.8

* Prevalence at enrolment i.e. after one year of stavudine containing ART.

¶ One or more of peripheral neuropathy, lipodystrophy, pancreatitis, hyperlactate syndrome, diabetes mellitus.

† severe hyperlactatemia, symptomatic hyperlactatemia and lactic acidosis, as defined in methods paragraph.

IR, incidence rate; PR, prevalence rate; CI, confidence interval; py, person-years; TC, total cholesterol serum level; TG, triglyceride serum level.

Elevated levels of total cholesterol and triglycerides were common but levels were generally only mildly raised. Dyslipidemia appeared to progress over time, reflected in a significant increase in the mean total cholesterol/HDL-cholesterol ratio (*table 3*). Fourteen patients (5.5%) changed from stavudine to zidovudine during follow up due to severe toxicity and in all except one, multiple side effects were present.

**Table 3:** Analysis of changes in serum lipid levels over time.

Lipids	n	month 12*	month 24	mean change	95% CI change	p-value
**Mean TC (mg/dL)**	209	159.5	163.9	4.4	−0.6–9.4	0.083
**% elevated TC (>200 mg/dL)**		12.1	21.1			0.1104
**Mean LDL-c (mg/dL)**	131	86.4	88.1	1.7	−2.5–6	0.42
**% elevated LDL-c (>130 mg/dL)**		5.3	7.6			0.4955
**Mean HDL-c (mg/dL)**	183	50.5	48.3	−2.2	−4.5–0.1	0.06
**% decreased HDL-c (<40 mg/dL)**		27.3	31.6			0.908
**Mean TG (mg/dL)**	202	96.5	103.3	6.6	−1.4–14.8	0.106
**% elevated TG (>110 mg/dL)**		29.5	37.6			0.005
**Mean TC/HDL-c ratio**	183	3.3	3.6	0.3	0.2–0.4	<0.001
**% elevated TC/HDL-c ratio (>5)**		5.5	9.8			0.121

TC, total cholesterol; LDL-c, low density lipoprotein cholesterol; HDL-c, high density lipoprotein cholesterol; TG, triglyceride.

* At the time of enrolment into the study, after 12 months of stavudine containing ART.

### Risk factors for common stavudine associated toxicities

Higher age was significantly associated with incident peripheral neuropathy, lipodystrophy and “any toxicity” in uni- and multivariate Cox regression analyses. Being in WHO clinical stage 3 or 4 (versus being in stage 1 or 2) at ART initiation was also independently associated with incident lipodystrophy (*table 4*).

**Table 4:** Cox regression models of variables associated with stavudine associated side effects.

Variable	Peripheral Neuropathy		Lipodystrophy		Any toxicity¶	
	HR (95%-CI)	aHR (95%-CI)	HR (95%-CI)	aHR (95%-CI)	HR (95%-CI)	aHR (95%-CI)
**Male gender**	1.26 (0.64–2.48)	1.67 (0.59–4.72)	0.73 (0.23–1.41)	0.73 (0.23–2.40)	1.05 (0.61–1.81)	1.08 (0.48–2.42)
**Age (years)**	**1.04 (1.02–1.08)**	**1.04 (1.00–1.08)**	**1.06 (1.03–1.11)**	**1.08 (1.04–1.13)**	**1.05 (1.02–1.08)**	**1.05 (1.02–1.08)**
**BMI (kg/m^2^)**	1.05 (0.99–1.14)	1.07 (0.97–1.18)	0.98 (0.87–1.10)	0.97 (0.84–1.12)	1.07 (1.00–1.15)	1.08 (0.99–1.17)
**WHO stage** *	1.36 (0.70–2.64)	1.68 (0.82–3.44)	0.52 (0.22–1.26)	**0.37 (0.14–0.98)**	0.95 (0.55–1.65)	0.96 (0.53–1.74)
**Alcohol use**	0.74 (0.10–5.74)	1.00 (0.13–7.85)			0.46 (0.06–3.31)	0.63 (0.08–4.79)
**TB diagnosis** †	0.65 (0.27–1.58)	0.61 (0.24–1.58)			0.92 (0.48–1.75)	1.15 (0.56–2.34)
**eCC (mL/min)**	1.00 (0.99–1.02)	0.99 (0.96–1.01)	0.98 (0.96–1.00)	0.99 (0.96–1.02)	1.00 (0.99–1.01)	0.99 (0.96–1.01)

¶ One or more of peripheral neuropathy, lipodystrophy, pancreatitis, high lactate syndrome, diabetes mellitus.

HR, hazard ratio; aHR, adjusted hazard ratio; CI, confidence interval, BMI, body mass index; WHO, world health organization; TB, tuberculosis;

eCC, estimate creatinine clearance.

* WHO stage refers to being in WHO clinic stage 3 or 4 vs. being in stage 1 or 2 at the start of ART. Significant associations are indicated in bold font.

† TB diagnosis refers to previous and current diagnosis.

## Discussion

We demonstrate a high prevalence of stavudine associated toxicities in a cohort of Malawian adults who completed the first year on ART and a high incidence of toxicities as they continued a stavudine based regimen. There was frequent occurrence of peripheral neuropathy and lipodystrophy, while high lactate syndromes and diabetes mellitus were rare and no pancreatitis was observed. Considerable toxicity related attrition from the standard regimen occurred, 10.5% replacing stavudine in the first year and 5.5% in the second.

Although the use of stavudine is declining in sub-Sahara Africa, this potent antiretroviral drug is still used by many adults in the region [13]. Good results have been achieved with stavudine based generic formulations, evidenced by low rates of treatment failure and switching to second-line regimens in observational studies from Mozambique [14], Cameroon [15], and Malawi [16]. In one of the few randomized controlled trials of ART regimens from Africa, a backbone of stavudine/lamivudine performed better than zidovudine/didanosine in terms of virological suppression and immune reconstitution, although toxicity was more severe [17].

As scale-up of ART progressed in sub-Saharan Africa, a number of cohort studies have highlighted the toxicity concerns of stavudine. Peripheral neuropathy (36%), but not pancreatitis (0.3%) and lactic acidosis (0.1%) were common in adult Ugandans starting stavudine based ART in a home-based care project in 2003–4 and being followed for a median of 11 months. Age ≥35 years and tuberculosis treatment were identified as risk factors for peripheral neuropathy [4]. A study from Kigali, Rwanda followed patients started on stavudine/lamivudine/nevirapine from 2003–7. Toxicity severe enough to replace stavudine was observed in 18.8%, mainly due to peripheral neuropathy (8.0%), lactic acidosis/symptomatic hyperlactatemia (3.1%) and lipodystrophy (7.2%). In this population, higher age and more advanced clinical stage were risk factors for peripheral neuropathy, female gender for lipodystrophy and higher weight and female gender for lactic acidosis/symptomatic hyperlactatemia [18]. In a large cohort from Johannesburg, South Africa, observed during 2004–8, moderate rates of peripheral neuropathy (17.1%) and lipodystrophy (7.4%) were found among those on stavudine. A large proportion (8.2%) experienced lactic acidosis/symptomatic hyperlactatemia. Women were at higher risk of all toxicities than men, except for peripheral neuropathy [5]. Although retention in care and virological control were good, substitution of stavudine was extremely common, especially among women [19].The same research group had earlier shown that stavudine substitutions were increased when ART was started with ongoing and concurrent TB treatment, but not in incident TB cases on established ART [20]. Female gender was also a major predictor of stavudine substitutions due to side effects in a cohort of adult Ugandans in Kampala [21].

These studies of stavudine toxicity are all limited by the fact that patients over 60 kg were exposed to a dosage of 40 mg per day. Since 2007 WHO has recommended dosing 30 mg of stavudine daily, irrespective of weight, as this is equally effective with a potentially lower rate of side effects [22]. Our study, in which 47.4% participants weighed >60 kg, shows that despite universal 30 mg dosing, stavudine associated toxicities are very common. The pattern of stavudine toxicities we found was largely comparable to the sub-Saharan studies described above. Our rate of lactic acidosis and symptomatic hyperlactatemia was somewhat lower than in some studies [5], [6] possibly because the patients we had enrolled had a relatively low mean BMI at the start of ART and we dosed 30 mg to heavier patients. We did not observe any cases of pancreatitis despite evaluating all suspected episodes. Others also found very few pancreatitis cases and because the epidemiology of pancreatitis in African populations is not well-known, it remains uncertain whether HIV and/or stavudine actually are risk factors. Dyslipidemia has been studied infrequently in ART patients from sub-Saharan Africa. Hypertriglyceridemia and hypercholesterolemia were present in 9% and 14% of adult Rwandans with lipodystrophy on mainly stavudine containing ART for at least 6 months. No hyperlipidemia was identified in patients without lipodystrophy [8]. Lipid profiles of rural Ugandans, studied at the start of stavudine containing ART and after 12 and 24 months, showed that mean total-, LDL- and HDL-cholesterol levels all increased over time, while mean triglyceride levels initially declined at 12 months, only to return to the baseline value after 24 months [23]. Although we did not have the benefit of pre-ART lipid values, over the course of the second year on ART we found trends of an increasing mean total cholesterol level and a decreasing mean HDL-cholesterol level, as well as a significantly increased mean total- to HDL-cholesterol ratio. Our data therefore suggest gradual atherogenic lipid changes with ongoing stavudine based ART. Further studies with longer follow up need to clarify whether stavudine induced lipid changes are associated with actual increased risk of cardiovascular disease.

We found that higher age was a risk factor for peripheral neuropathy as was the case in other studies [4], [17] and for lipodystrophy and “any stavudine toxicity”. This suggests that older patients have to be considered as a priority group qualifying for a non-stavudine containing standard first-line regimen, as is currently the case in Malawi with children, breastfeeding and pregnant women and TB patients [24].

Strengths of our study are that we comprehensively describe the full array of stavudine associated toxicities and that we had low losses to follow up. Limitations also need to be considered. We enrolled patients who had remained on stavudine containing ART in the clinic one year after starting treatment. For a complete picture of stavudine toxicities in the first year, events in all patients who started stavudine/lamivudine/nevirapine at the same time as our participants and were not enrolled needed to be regarded. However description of events in these patients depended on retrospective analysis of routine data from patient records and the electronic database of the clinic. This may have resulted in a less complete and accurate overview of stavudine side effects in the first year on ART. Although we have assumed that the described adverse effects were toxicities of stavudine, it cannot be excluded that in some cases other factors were important, such as lamivudine toxicity and impact of the virus itself. Comparison of toxicities in patients who are not on stavudine are not available from Malawi, because all patients have started the same first-line regimen since the commencement of the scale up programme. Only very recently has a new first-line regimen, consisting of tenofovir, lamivudine and efavirenz been introduced for certain priority groups. We did not use some objective measures (waist-to-hip ration) and methods (DEXA scanning) for the determination of lipodystrophy. Our study was done in an urban rather than a rural clinic which must be considered when extrapolating to other settings.

In times of economic austerity, it is crucial for donor funded programmes to continue using standardized, affordable first-line regimens, so as to avoid treatment discontinuations due to drug stock outs, since this is associated with interruption of care and with death [25]. In a computer model of HIV treatment in RSA, stavudine/lamivudine/nevirapine was the least cost-effective of five regimens considered (despite the lower drug price) and tenofovir/lamivudine/efavirenz the most cost-effective [10]. However considerable initial investments for an alternative to stavudine are needed. This appears justifiable given the high rates of toxicity we and others recorded. Stavudine toxicities obviously cause direct harm to patients, also have the potential to increase the rate of cardio- and cerebro-vascular disease, and increasingly attract national media attention, particularly in relation to lipodystrophy [26], together affecting the reputation of ART in general. Our data provide further compelling evidence for the donor community and national governments to prioritize the replacement of stavudine in the standard first-line regimen for adults in sub-Saharan Africa.
